# Linoleic acid and linoleate diols in neonatal cord blood influence birth weight

**DOI:** 10.3389/fendo.2022.986650

**Published:** 2022-08-25

**Authors:** Naoko Umeda, Takaharu Hirai, Takayo Ohto-Nakanishi, Kenji J. Tsuchiya, Hideo Matsuzaki

**Affiliations:** ^1^ Department of Functional Brain Activities, United Graduate School of Child Development, Hamamatsu University School of Medicine, Osaka University, Kanazawa University, Chiba University, and University of Fukui, Osaka, Japan; ^2^ Research Center for Child Mental Development, University of Fukui, Fukui, Japan; ^3^ Department of Nursing, Faculty of Health Science, Fukui Health Science University, Fukui, Japan; ^4^ Department of Psychiatric and Mental Health Nursing, School of Nursing, University of Fukui, Fukui, Japan; ^5^ Lipidome Lab Co., Ltd., Akita, Japan; ^6^ Research Center for Child Mental Development, Hamamatsu University School of Medicine, Shizuoka, Japan; ^7^ Life Science Innovation Center, University of Fukui, Fukui, Japan

**Keywords:** epoxy fatty acids, soluble epoxide hydrolase, cytochrome P450, low birth weight (LBW), linoleic acid, linoleate diol

## Abstract

**Background:**

Low-birth-weight infants exhibit a high risk for postnatal morbidity. Cytochrome P450 (CYP) and epoxide hydrolase (EH) are involved in the metabolism of factors responsible for low-birth-weight in infants. Both CYPs and EHs have high substrate specificity and are involved in polyunsaturated fatty acid (PUFA) metabolism. The CYP pathway produces epoxy fatty acids (EpFAs), which are further degraded by soluble EH (sEH). Additionally, sEH inhibition enhances the action of EpFAs and suppresses inflammatory responses. During pregnancy, excessive activation of maternal inflammatory response is a significant factor associated with low-birth-weight. However, the association of EpFAs, which have potential anti-inflammatory properties, with the low-birth-weight of infants remains uninvestigated. This study aimed to clarify the association between the umbilical cord serum EpFA and low-birth-weight using data obtained from the Hamamatsu Birth Cohort for Mothers and Children (HBC Study) by analyzing the umbilical cord blood samples.

**Method:**

We selected a subgroup of 200 infants (106 boys and 94 girls), quantified EpFA concentration in their cord blood samples collected at birth, and examined its correlation with birth weight.

**Results:**

The comparison between the low-birth-weight and normal-birth-weight groups revealed no significant correlation between PUFA and EpFA concentrations, but a significant correlation was observed in the linoleate diol concentrations of the two groups. Furthermore, birth weight did not significantly correlate with PUFA, EpFA, and diol concentrations in cord blood; however, multiple regression analysis showed a significant negative correlation of birth weight with the concentration of linoleic acid (LA) (*r* = −0.101, *p* = 0.016) as well as LA-derived dihydroxyoctadecenoic acid (diHOME) (*r* = −0.126, *p* = 0.007), 9,10-diHOME (*r* = −0.115, *p* = 0.014), and 12,13-diHOME (*r* = −0.126, *p* = 0.007) after adjusting for obstetric factors, including gestational age, infant’s sex, childbirth history, delivery method, and maternal height.

**Conclusions:**

Birth weight was significantly correlated with the concentration of LA and linoleate diol diHOME after adjusting for obstetric confounders. Our results show that CYP and sEH involved in PUFA metabolism may influence the birth weight of infants. Further validation is needed to provide insights regarding maternal intervention strategies required to avoid low-birth-weight in infants in the future.

## Introduction

The World Health Organization defines a low-birth-weight infant as an infant weighing <2500 g regardless of the gestational age. The birth weight of infants has been declining annually, and low-birth-weight infants exhibit high postnatal morbidity and mortality (1). Low-birth-weight is a powerful predictor of infant survival and childhood morbidity as well as adulthood health conditions (1). The proposed causes of low-birth-weight include several environmental factors, such as maternal anemia (2, 3) lipid and sugar metabolism abnormalities (4, 5), alcohol consumption (6), caffeine consumption (7–9), acrylamide exposure (10), tobacco smoking (11–14), and pesticide exposure (15, 16). It is well known that estrogen and progesterone regulate the conception and continuation of pregnancy (17), but recently, the attention has shifted to the dependency of pregnancy on the regulation of immune processes in the placenta (18). The important mediators of an immune response include pro- and anti-inflammatory interleukins, which are potentially regulated by cytochrome P450 (CYP), CYP epoxygenase, soluble epoxide hydrolase (sEH), and their metabolites (19, 20). Although the cause of low-birth-weight remains unclear, recent research has focused on CYP and EH, which are involved in the metabolism of the abovementioned factors. Maternal caffeine intake during pregnancy and *CYP1A2* C164A polymorphism affect infant birth size (7). Prenatal exposure to dietary acrylamide has been reported to be inversely associated with birth weight *via* interaction patterns with SNPs in *CYP2E1* and EH 1 (*EPHX1*) (10). Additionally, *CYP1A1* and *EPHX1* influence the association between maternal passive smoking and birth weight (21). Higher levels of organochlorine pesticide and the A1A1 genotype of *CYP17A1* in pregnant women may be important factors for idiopathic small-for-gestational-age (SGA) infants (16). CYPs are found in the endoplasmic reticulum and mitochondria of cells and are involved in various reactions necessary for normal biological activities, including liver detoxification, steroid hormone biosynthesis, and fatty acid metabolism. EH participates in compound metabolism and produces diol products through epoxide hydrolysis reactions. Both CYPs and EHs have high substrate specificity and are involved in the metabolism of polyunsaturated fatty acids (PUFA), including ω6 fatty acids (linoleic acid [LA] and arachidonic acid [AA]) and ω3 fatty acids (eicosapentaenoic acid [EPA] and docosahexaenoic acid [DHA]); these fatty acids are important for maintaining normal physiological functions (22–24). Most PUFAs are metabolized *via* three pathways. The cyclooxygenase (COX) and lipoxygenase (LOX) pathways primarily produce inflammatory metabolites (25), whereas the CYP pathway produces epoxy fatty acids (EpFAs), which are further degraded by sEH. With microvasculature maintenance and mitochondrial and endoplasmic reticulum stress inhibition, EpFA can yield extremely potent anti-inflammatory and antioxidant effects (26). In animal models of inflammatory diseases, sEH inhibition enhances EpFA action and suppresses inflammatory responses (27, 28). During pregnancy, excessive activation of maternal inflammatory response is a significant factor associated with low-birth-weight in infants; additionally, the placenta has high CYP concentrations, and the AA metabolism in the CYP pathway affects fetal development (18, 29–35). However, the association of EpFAs having potent anti-inflammatory properties with low-birth-weight remains unreported.

We hypothesized that the reduction in the levels of specific EpFA might be responsible for low-birth-weight in infants. Hence, this study aimed to clarify the association between umbilical cord serum EpFA and birth weight using data obtained from the Hamamatsu Birth Cohort for Mothers and Children (HBC) Study by analyzing umbilical cord blood serum samples through liquid chromatography coupled with tandem mass spectrometry (LC–MS/MS) according to Lipidome Lab Co., Ltd.

## Materials and methods

### Participants

This study was conducted as a part of the HBC Study. The details of the cohort setting have been previously reported (36, 37). Originally, the cohort comprised 1138 mothers who delivered between December 2007 and March 2012 and 1258 children born to them. Their demographic and perinatal data were comparable to those of mothers and children in the general population of Japan (36). In this study, we selected a subgroup out of the original cohort. This subgroup comprised 200 children whose cord blood samples collected at birth were available. Further, premature, and multiple births were excluded due to obstetric factors. Fatty acid concentrations in cord blood collected at birth were quantified and their correlation with birth weight was determined.

### Ethics statement

In accordance with the Declaration of Helsinki, the study was approved by the Ethics Review Committee of the Hamamatsu University School of Medicine (Nos. 20–82, 21–114, 22–29, 24–67, 24–237, 25–143, 25–283, E14–062, 17–037, 17–037-3, and 20–233) and the Research Ethics Committee of the University of Fukui. All mothers provided written informed consent for their and their children’s participation in the study.

### LC–MS/MS analysis of EpFA in the umbilical cord blood

Immediately after delivery, the umbilical cord blood samples were collected from the mothers *via* the venipuncture of the umbilical vein. Subsequently, the samples were kept at room temperature for 30 min and centrifuged at 3500 rpm for 10 min, from which serum was collected and divided into 200 μL aliquots, and then stored at −80°C until analysis (37).

The lipid fraction containing EpFA was isolated from 180 µL of serum through solid phase extraction with Oasis HLB columns (Waters Corporation, MA, USA). EpFA was separated using a high-performance liquid chromatography system (Nexera LC-30AD, Shimadzu Corporation, Kyoto, Japan) equipped with an XBridge C18 column (particle size, 3.5 µm; length, 150 mm; inner diameter, 1.0 mm; Waters) and analyzed on a triple quadrupole mass spectrometer (LC-MS-8040; Shimadzu). Mass spectrometric analysis was conducted in negative-ion mode, and fatty acid metabolites were identified and quantified by multiple-reaction monitoring, similar to the determination of other lipid metabolites (38). For quantification, calibration curves were prepared for each compound and recoveries were monitored using the deuterated internal standard ((±)11 (12)-epoxyeicosatrienoic acid-d11 [11,12-EET-d11], (±)12 (13)-dihydroxyoctadecenoic acid [12,13-diHOME-d4] and arachidonic acid-d8 [AA-d8]; Cayman Chemicals, Ann Arbor, Michigan, USA). Data were analyzed using LabSolutions software (Shimadzu), and LC–MS/MS analysis was conducted according to Lipidome Lab Co., Ltd.

The analytical values lesser than the lower limit of quantitation and detection were below the minimum reliable values for concentration measurement. Therefore, any value below the detection limit was treated as “0” and included in our analysis.

### Multiplex assay

Serum levels of interleukins were assayed using a suspension array system (Bio-Plex; Bio-Rad, Hercules, CA, USA). Multiplex kits for measuring interleukins were purchased from Bio-Rad (Bio-Plex Pro Human Cytokine 27-Plex Panel; Bio-Rad, Hercules, CA). The kits were used per the manufacturer’s instructions. The serum samples were diluted using the appropriate sample diluents provided in each kit in accordance with the manufacturer’s instructions. Concentrations (pg/ml) of different analytes in the serum samples were determined by using the standard curves generated in the multiplex assays. Each standard curve was generated using eight points of concentrations, and a nonlinear least squares minimization algorithm was used for the curve fitting by the five-parameter logistic equation and to determine the high and low limits of detection. Data points for analytes that were occasionally above or below the detection range were discarded.

### Perinatal variables

Data regarding weight at birth, gestational age at birth, sex, and parity of the child were collected from the medical records in the HBC Study. Furthermore, the participating children were divided into the SGA and appropriate-for-gestational age (AGA) groups based on the representative statistics calculated by the Japanese Society of Gynecology and Obstetrics (39). The infants above the upper limit of AGA, i.e., large-for-gestational age, were included in the AGA group for ease of interpretation.

### Statistical analyses

Mann–Whitney *U* test was used to compare birth weights between the SGA and AGA groups, and Spearman’s correlation was used to analyze the correlations between birth weight and fatty acids. Birth weight was regressed onto the PUFA metabolites, with gestational age at birth, infant’s sex, parity, delivery method (cesarean section), and maternal height as potential confounders according to previous studies (40–42). All data were analyzed using SPSS Statistics version 28 (IBM, Armonk, NY, USA).

## Results

### Participant characteristics

Among the 200 children enrolled in this study, 106 were boys and 94 were girls (**Table 1**). Of these, six twins (three boys, three girls) and four preterm births (two boys, two girls) were excluded. Ultimately, 190 participants (101 boys, 89 girls) were included in the analysis. The mean maternal age at birth, gestational age at birth, and birth weight of infants were 32.21 (standard deviation [SD], 4.98 [range: 19.0–43.4]) years, 39.22 (SD, 1.13 [range: 37.00–41.85]) weeks, and 3029 g (SD, 370.5 g [1930–4170 g]), respectively (**Table 1**). Thirteen participants (6.8%) had a low-birth-weight (<2500 g) and 11 (5.7%) were categorized as SGA (**Table 1**).

**Table 1:** Maternal and neonatal background characteristics.

	Mean (SD) or n (%)	Range
n = 190 **Maternal**		
Age (years)	32.21 (SD 4.98)	19.0 - 43.4
Height (cm)	158.0 (SD 5.62)	142.0 - 173.0
Vaginal delivery	154 (81.0%)	
Cesarean section	36 (19.0%)	
Primipara	96 (50.5%)	
Multipara	94 (49.5%)	
Smoking history	28 (14.7%)	
Smoking during pregnancy	10 (5.3%)	
**Infant**		
Gestational age (weeks)	39.22 (SD 1.13)	37.0-41.85
Sex		
Male	101	
Female	89	
Birth weight (g)	3029 (SD 370.5)	1930-4170
Low-birth-weight (<2500 g)	13 (6.8%)	
SGA	11 (5.7%)	

SD, standard deviation; SGA, small for gestational age.

### PUFA metabolite profile in cord blood

The mean concentrations of ω6 fatty acids LA and AA were 3.061 × 10^6^ pg/mL (SD, 2.174 × 10^6^ pg/mL [0.297 × 10^6^–10.59 × 10^6^ pg/mL]) and 2.060 × 10^5^ pg/mL (SD, 0.768 × 10^5^ pg/mL [0.683 × 10^5^–4.339 × 10^5^ pg/mL]), respectively (**Table 2**). For EPA and DHA, which are ω3 fatty acids, the mean concentrations were 1.749 × 10^4^ pg/mL (SD, 0.761 × 10^4^ pg/mL [0.524 × 10^4^–5.297 × 10^4^ pg/mL]) and 7.795 × 10^5^ pg/mL (SD, 4.737 × 10^5^ pg/mL [2.230 × 10^5^–27.19 × 10^5^ pg/mL]), respectively (**Table 2**).

**Table 2:** PUFA metabolite profile of cord blood.

	LLOQ (n)	LLOD (n)	Mean	SD	Range		
**LA**	0	0	3.061 × 10^6^	2.174 × 10^6^	0.297 × 10^6^	–	10.59 × 10^6^
EpOME			1.087 × 10^3^	0.734 × 10^3^	0.399 × 10^3^	–	6.633 × 10^3^
9,10-EpOME	26	1	0.476 × 10^3^	0.401 × 10^3^	0.000	–	3.546 × 10^3^
12,13-EpOME	7	1	0.610 × 10^3^	0.348 × 10^3^	0.000	–	3.086 × 10^3^
diHOME			1.310 × 10^3^	0.474 × 10^3^	0.641 × 10^3^	–	4.774 × 10^3^
9,10-diHOME	0	0	0.446 × 10^2^	0.252 × 10^2^	0.119 × 10^2^	–	1.938 × 10^2^
12,13-diHOME	0	0	1.265 × 10^3^	0.450 × 10^3^	0.620 × 10^3^	–	4.580 × 10^3^
**AA**	0	0	2.060 × 10^5^	0.768 × 10^5^	0.683 × 10^5^	–	4.339 × 10^5^
EET			0.382 × 10^3^	0.464 × 10^3^	0.000	–	3.792 × 10^3^
5,6-EET	20	96	0.109 × 10^3^	0.166 × 10^3^	0.000	–	1.294 × 10^3^
8,9-EET	76	100	0.179 × 10^3^	0.220 × 10^3^	0.000	–	1.231 × 10^3^
11,12-EET	89	70	0.865 × 10^2^	0.849 × 10^2^	0.000	–	6.930 × 10^2^
14,15-EET	21	124	0.713 × 10^2^	1.048 × 10^2^	0.000	–	7.405 × 10^2^
diHETrE			3.470 × 10^3^	0.949 × 10^3^	1.466 × 10^3^	–	6.697 × 10^3^
5,6-diHETrE	25	75	0.226 × 10^3^	0.124 × 10^3^	0.096 × 10^3^	–	0.898 × 10^3^
8,9-diHETrE	0	0	1.113 × 10^3^	0.413 × 10^3^	0.319 × 10^3^	–	2.369 × 10^3^
11,12-diHETrE	0	0	0.844 × 10^3^	0.292 × 10^3^	0.312 × 10^3^	–	1.841 × 10^3^
14,15-diHETrE	0	0	1.377 × 10^3^	0.397 × 10^3^	0.294 × 10^3^	–	2.574 × 10^3^
**EPA**	0	0	1.749 × 10^4^	0.761 × 10^4^	0.524 × 10^4^	–	5.297 × 10^4^
EpETE			3.693	29.362	0.000	–	2.602 × 10^2^
5,6-EpETE	0	190	0.000	0.000	0.000	–	0.000
8,9-EpETE	2	188	3.087	26.064	0.000	–	2.351 × 10^2^
11,12-EpETE	0	190	0.000	0.000	0.000	–	0.000
14,15-EpETE	0	190	0.000	0.000	0.000	–	0.000
17,18-EpETE	1	189	1.820	21.760	0.000	–	2.602 × 10^2^
diHETE			6.280 × 10^3^	3.124 × 10^3^	0.696 × 10^3^	–	29.90 × 10^3^
5,6-diHETE	0	190	0.000	0.000	0.000	–	0.000
8,9-diHETE	21	165	3.402 × 10^2^	1.478 × 10^2^	1.742 × 10^2^	–	8.121 × 10^2^
11,12-diHETE	2	38	1.243 × 10^2^	5.018 × 10^2^	3.350 × 10^2^	–	4.155 × 10^2^
14,15-diHETE	50	38	6.958 × 10^2^	2.834 × 10^2^	1.600 × 10^2^	–	22.25 × 10^2^
17,18-diHETE	0	1	5.609 × 10^3^	2.844 × 10^3^	1.674 × 10^3^	–	28.21 × 10^3^
**DHA**	1	0	7.795 × 10^5^	4.737 × 10^5^	2.230 × 10^5^	–	27.19 × 10^5^
EpDPA			3.311 × 10^2^	6.128 × 10^2^	0.000	–	50.27 × 10^2^
4,5-EpDPA	0	190	0.000	0.000	0.000	–	0.000
7,8-EpDPA	9	176	0.309 × 10^2^	1.026 × 10^2^	0.000	–	5.574 × 10^2^
10,11-EpDPA	87	52	1.170 × 10^2^	1.015 × 10^2^	0.000	–	6.363 × 10^2^
13,14-EpDPA	3	183	0.200 × 10^2^	0.964 × 10^2^	0.000	–	6.300 × 10^2^
16,17-EpDPA	31	157	0.465 × 10^2^	1.272 × 10^2^	0.000	–	11.37 × 10^2^
19,20-EpDPA	18	148	1.956 × 10^2^	4.966 × 10^2^	0.000	–	36.54 × 10^2^
diHDoPE			5.749 × 10^3^	1.916 × 10^3^	2.159 × 10^3^	–	15.46 × 10^3^
4,5-diHDoPE			N/A	N/A	N/A		N/A
7,8-diHDoPE	41	140	4.164 × 10^2^	2.690 × 10^2^	2.209 × 10^2^	–	2.074 × 10^3^
10,11-diHDoPE	1	3	2.564 × 10^2^	1.143 × 10^2^	0.581 × 10^2^	–	8.108 × 10^2^
13,14-diHDoPE	0	0	8.993 × 10^2^	3.156 × 10^2^	2.340 × 10^2^	–	18.30 × 10^2^
16,17-diHDoPE	0	3	6.481 × 10^2^	1.960 × 10^2^	2.221 × 10^2^	–	12.34 × 10^2^
19,20-diHDoPE	0	0	3.850 × 10^3^	1.331 × 10^3^	1.145 × 10^3^	–	11.28 × 10^3^

PUFA, polyunsaturated fatty acid; LLOQ, lower limit of quantitation; LLOD, lower limit of detection; SD, standard deviation; LA, linoleic acid; EpOME, epoxyoctadecamonoenoic acid; diHOME, dihydroxyoctadecenoic acid; AA, arachidonic acid; EET, epoxyeicosatrienoic acid; diHETrE, dihydroxyeicosaterolaenoic acid; EPA, eicosapentaenoic acid; EpETE, epoxyeicosatetraenoic acid; diHETE dihydroxyeicosatrienoic acid; DHA, docosahexaenoic acid; EpDPA, epoxydocosapentaenoic acid; diHDoPE, dihydroxydocosapentaenoic acid; N/A, not applicable.

The concentrations of EpFAs metabolized from PUFA were quantified. For instance, the mean concentration of total epoxy octadecenoic acid (EpOME) from LA was 1.087 × 10^3^ pg/mL (SD, 0.734 × 10^3^ pg/mL [0.399 × 10^3^–6.633 × 10^3^ pg/mL]) (**Table 2**). The mean concentration of total epoxy eicosatrienoic acid (EET) from AA was 0.382 × 10^3^ pg/mL (SD, 0.464 × 10^3^ pg/mL [0–3.792 × 10^3^ pg/mL]); however, AA-derived EET was 0 pg/mL in 51 samples (**Table 2**). The mean concentration of total epoxy eicosatetraenoic acid (EpETE) from EPA was 3.693 pg/mL (SD, 29.36 pg/mL [0–260.2 pg/mL]), but EPA-derived EpETE was 0 pg/mL in 187 samples (**Table 2**). Furthermore, the mean concentration of total epoxy docosapentaenoic acid (EpDPA) from DHA was 3.311 × 10^2^ pg/mL (SD, 6.128 × 10^2^ pg/mL [0–50.27 × 10^2^ pg/mL]), but DHA-derived EpDPA was 0 pg/mL in 44 samples (**Table 2**).

The concentrations of diols metabolized by sEH were quantified. The mean concentration of total dihydroxyoctadecenoic acid (diHOME) from LA was 1.310 × 10^3^ pg/ml (SD, 0.474 × 10^3^ pg/ml [0.641 × 10^3^–4.774 × 10^3^ pg/ml]) (**Table 2**). The mean concentration of dihydroxyeicosaterolaenoic acid (diHETrE) from AA was 3.470 × 10^3^ pg/ml (SD, 0.949 × 10^3^ pg/ml ([1.466 × 10^3^–6.697 × 10^3^ pg/ml]) (**Table 2**). The mean concentration of dihydroxyeicosatrienoic acid (diHETE) from EPA was 6.280 × 10^3^ pg/ml (SD, 3.124 × 10^3^pg/ml [0.696 × 10^3^–29.90 × 10^3^ pg/ml]) (**Table 2**). The mean concentration of dihydroxydocosapentaenoic acid (diHDoPE) from DHA was 5.749 × 10^3^ pg/ml (SD, 1.916 × 10^3^ pg/ml [2.159 × 10^3^–15.46 × 10^3^ pg/ml]) (**Table 2**).

### Correlation between birth weight and PUFA metabolite in cord blood

Birth weight was strongly correlated with maternal factors, such as gestational age at birth (*r =* 0.323, *p <* 0.001, 95% confidence interval [CI] = 0.185–0.448) and maternal height (*r =* 0.326, *p <* 0.001, 95% CI = 0.189–0.451) (**Table 3**). However, no correlation was found between maternal age and gestational age at birth (*r =* 0.046, *p =* 0.531, 95% CI = −0.101–0.191) (**Table 3**). None of the fatty acids, such as LA (*r =* −0.099, *p =* 0.175), AA (*r =* 0.071, *p =* 0.330), EPA (*r =* 0.072, *p =* 0.324), and DHA (*r =* −0.055, *p =* 0.449), showed significant correlations with birth weight (**Table 3**). Likewise, birth weight demonstrated no significant correlation with EpOME (*r =* 0.033, *p =* 0.651), EET (*r =* 0.012, *p =* 0.865), and EpDPA (*r =* −0.084, *p =* 0.251). diHOME (*r* = −0.132, *p* = 0.069), diHETrE (*r* = −0.050, *p* = 0.494), diHETE (*r* = −0.109, *p* = 0.135) and diHDoPE (*r* = −0.041, *p* = 0.572) also showed no significant correlation with birth weight (**Table 3**). Only the total EpETE concentration showed a significant correlation with birth weight (r = 0.143, p = 0.049) (**Table 3**).

**Table 3:** Correlation between birth weight and PUFA metabolite in cord blood.

		Birth weight n = 190				
		*r*	*p* value	95% CI		
**Maternal**						
	Age(years)	0.046	0.531	−0.101	–	0.191
	Height (cm)	0.326	<0.001	0.189	–	0.451
**Infant**						
	Gestational age (weeks)	0.323	<0.001	0.185	–	0.448
					
**LA**		−0.099	0.175	−0.242	–	0.049
Total EpOME		0.033	0.651	−0.114	–	0.179
	9,10-EpOME	0.060	0.407	−0.087	–	0.205
	12,13-EpOME	−0.001	0.986	−0.148	–	0.145
Total diHOME		−0.132	0.069	−0.273	–	0.015
	9,10-diHOME	−0.130	0.075	−0.271	–	0.017
	12,13-diHOME	−0.131	0.072	−0.272	–	0.016
						
**AA**		0.071	0.330	−0.076	–	0.215
Total EET		0.012	0.865	−0.134	–	0.159
	5,6-EET	0.136	0.062	−0.011	–	0.278
	8,9-EET	−0.055	0.497	−0.217	–	0.109
	11,12-EET	−0.034	0.665	−0.191	–	0.124
	14,15-EET	−0.009	0.911	−0.174	–	0.156
diHETrE		−0.050	0.494	−0.195	–	0.097
	5,6-diHETrE	−0.065	0.493	−0.250	–	0.125
	8,9-diHETrE	−0.114	0.117	−0.256	–	0.033
	11,12-diHETrE	0.009	0.906	−0.138	–	0.155
	14,15-diHETrE	−0.002	0.978	−0.148	–	0.145
						
**EPA**		0.072	0.324	−0.075	–	0.216
Total EpETE		0.143	0.049	−0.004	–	0.283
	5,6-EpETE	N/A	N/A	N/A		N/A
	8,9-EpETE	0.101	0.229	−0.069	–	0.266
	11,12-EpETE	N/A	N/A	N/A		N/A
	14,15-EpETE	N/A	N/A	N/A		N/A
	17,18-EpETE	0.124	0.140	−0.046	–	0.287
diHETE		−0.109	0.135	−0.251	–	0.038
	5,6-diHETE	N/A	N/A	N/A		N/A
	8,9-diHETE	−0.041	0.847	−0.439	–	0.371
	11,12-diHETE	−0.057	0.488	−0.218	–	0.108
	14,15-diHETE	−0.079	0.336	−0.239	–	0.086
	17,18-diHETE	−0.116	0.113	−0.258	–	0.032
						
**DHA**		−0.055	0.449	−0.200	–	0.092
Total EpDPA		−0.084	0.251	−0.227	–	0.064
	4,5-EpDPA	N/A	N/A	N/A		N/A
	7,8-EpDPA	−0.038	0.653	−0.204	–	0.131
	10,11-EpDPA	−0.053	0.499	−0.210	–	0.106
	13,14-EpDPA	−0.022	0.796	−0.189	–	0.147
	16,17-EpDPA	0.041	0.622	−0.127	–	0.208
	19,20-EpDPA	−0.154	0.059	−0.310	–	0.010
diHDoPE		−0.041	0.572	−0.187	–	0.106
	4,5-diHDoPE					
	7,8-diHDoPE	0.026	0.859	−0.262	–	0.310
	10,11-diHDoPE	−0.039	0.599	−0.185	–	0.110
	13,14-diHDoPE	−0.031	0.675	−0.176	–	0.116
	16,17-diHDoPE	−0.030	0.682	−0.177	–	0.118
	19,20-diHDoPE	−0.039	0.593	−0.184	–	0.108

PUFA, polyunsaturated fatty acid; 95% CI, 95% confidence interval; LA, linoleic acid; EpOME, epoxyoctadecamonoenoic acid; diHOME, dihydroxyoctadecenoic acid; AA, arachidonic acid; EET, epoxyeicosatrienoic acid; diHETrE, dihydroxyeicosaterolaenoic acid; EPA, eicosapentaenoic acid; EpETE, epoxyeicosatetraenoic acid; diHETE dihydroxyeicosatrienoic acid; DHA, docosahexaenoic acid; EpDPA, epoxydocosapentaenoic acid; diHDoPE, dihydroxydocosapentaenoic acid; N/A, not applicable.

### Correlation between interleukin levels in cord blood and birth weight

In terms of inflammatory metabolites, we examined the association between interleukin concentrations in cord blood and birth weight, but the correlations were not significant (**Table 4**).

**Table 4:** Interleukin concentration (pg/ml) in cord blood and its correlation with birth weight.

								Birth weight n = 156				
	LLOQ (n)	LLOD (n)	Average	SD	Range			*r*	*p value*	95% CI		
Interleukin-1α	26	0	3.748	1.909	0.700	–	8.053	0.056	0.485	−0.106	–	0.216
Interleukin-1β	27	0	7.786	3.383	2.100	–	13.920	−0.038	0.638	−0.198	–	0.125
Interleukin-2	11	0	5.723	1.656	1.400	–	9.820	−0.082	0.311	−0.240	–	0.081
Interleukin-4	114	0	1.535	0.753	1.100	–	3.755	−0.053	0.511	−0.213	–	0.110
Interleukin-5	6	0	2.243	0.582	0.400	–	4.077	−0.034	0.676	−0.194	–	0.129
Interleukin-6	129	0	5.624	17.475	2.400	–	161.033	−0.042	0.600	−0.203	–	0.120
Interleukin-8	0	0	13.000	14.385	2.127	–	107.910	−0.011	0.891	−0.172	–	0.151
Interleukin-10	65	0	5.315	7.267	1.800	–	74.203	0.022	0.784	−0.140	–	0.183
Interleukin-12p70	39	0	2.852	1.192	1.200	–	6.685	0.098	0.224	−0.065	–	0.256
Interleukin-13	37	0	2.269	1.123	0.700	–	5.817	−0.008	0.918	−0.170	–	0.154
Interleukin-15	95	0	3.125	1.986	1.700	–	8.825	−0.002	0.985	−0.163	–	0.160
Interleukin-17	142	0	1.565	0.533	1.400	–	3.557	0.109	0.176	−0.054	–	0.266
Interleukin-23	3	0	25.718	4.448	5.000	–	33.513	0.038	0.639	−0.125	–	0.198

LLOQ, lower limit of quantitation; LLOD, lower limit of detection; SD, standard deviation; 95% CI, 95% confidence interval.

### Correlation between gestational age and fatty acid metabolite in cord blood

None of the fatty acids, including LA (*r* = −0.089, *p* = 0.223), AA (*r* = 0.051, *p* = 0.485), EPA (*r* = 0.053, *p* = 0.470), and DHA (*r* = −0.055, *p* = 0.454), showed significant correlations with gestational age (**Table 5**). Likewise, gestational age demonstrated no significant correlation with EpOME (*r* = 0.027, *p* = 0.710), EET (*r* = 0.010, *p* = 0.886), and EpDPA (r = −0.090, p = 0.216) (**Table 5**). Total diHOME (*r* = 0.061, *p* = 0.407), diHETrE (*r* = 0.019, *p* = 0.794), diHETE (*r* = −0.063, *p* = 0.391), and diHDoPE (*r* = 0.024, *p* = 0.744) also showed no significant correlation (**Table 5**). Only 11,12-EET and total EpETE concentrations showed a significant correlation with gestational age (11,12-EET: *r* = −0.166, *p* = 0.033; total EpETE: *r* = 0.165, *p* = 0.023) (**Table 5**).

**Table 5:** Correlation between gestational age and PUFA metabolite in cord blood.

		GA n = 190				
		*r*	*p value*	95%CI		
**Maternalternal**						
	Age (years)	−0.185	0.011	−0.323	–	−0.039
	Height (cm)	−0.042	0.567	−0.187	–	0.105
	Cesarean section	−0.401	<0.001	−0.517	–	−0.271
**Infant**						
	Sex	−0.039	0.591	−0.185	–	0.108
	Birth weight	0.323	<0.001	0.185	–	0.448
						
**LA**		−0.089	0.223	−0.232	–	0.058
Total EpOME		0.027	0.710	−0.120	–	0.173
	9,10-EpOME	0.000	0.996	−0.146	–	0.147
	12,13-EpOME	0.050	0.493	−0.097	–	0.195
Total diHOME		0.061	0.407	−0.087	–	0.205
	9,10-diHOME	0.064	0.380	−0.083	–	0.209
	12,13-diHOME	0.060	0.414	−0.088	–	0.204
						
**AA**		0.051	0.485	−0.096	–	0.196
Total EET		0.010	0.886	−0.136	–	0.157
	5,6-EET	−0.045	0.536	−0.191	–	0.102
	8,9-EET	0.036	0.656	−0.128	–	0.199
	11,12-EET	−0.166	0.033	−0.316	–	−0.009
	14,15-EET	−0.042	0.611	−0.206	–	0.124
Total diHETrE		0.019	0.794	−0.128	–	0.165
	5,6-diHETrE	−0.024	0.797	−0.212	–	0.165
	8,9-diHETrE	−0.060	0.414	−0.204	–	0.088
	11,12-diHETrE	−0.007	0.920	−0.154	–	0.139
	14,15-diHETrE	0.060	0.414	−0.088	–	0.204
						
**EPA**		0.053	0.470	−0.095	–	0.198
Total EpETE		0.165	0.023	0.019	–	0.304
	5,6-EpETE	N/A	N/A	N/A		N/A
	8,9-EpETE	0.130	0.121	−0.039	–	0.293
	11,12-EpETE	N/A	N/A	N/A	–	N/A
	14,15-EpETE	N/A	N/A	N/A	–	N/A
	17,18-EpETE	0.126	0.133	−0.044	–	0.289
Total diHETE		−0.063	0.391	−0.207	–	0.085
	5,6-diHETE	N/A	N/A	N/A	–	N/A
	8,9-diHETE	−0.017	0.934	−0.420	–	0.391
	11,12-diHETE	−0.074	0.366	−0.235	–	0.091
	14,15-diHETE	−0.030	0.714	−0.193	–	0.134
	17,18-diHETE	−0.078	0.285	−0.222	–	0.070
						
**DHA**		−0.055	0.454	−0.200	–	0.093
Total EpDPA		−0.090	0.216	−0.234	–	0.057
	4,5-EpDPA	N/A	N/A	N/A		N/A
	7,8-EpDPA	−0.055	0.508	−0.221	–	0.113
	10,11-EpDPA	−0.123	0.118	−0.276	–	0.036
	13,14-EpDPA	−0.028	0.735	−0.196	–	0.141
	16,17-EpDPA	−0.055	0.513	−0.220	–	0.114
	19,20-EpDPA	−0.088	0.279	−0.249	–	0.077
Total diHDoPE		0.024	0.744	−0.123	–	0.170
	4,5-diHDoPE	N/A	N/A	N/A	–	N/A
	7,8-diHDoPE	−0.118	0.414	−0.391	–	0.174
	10,11-diHDoPE	−0.061	0.405	−0.207	–	0.087
	13,14-diHDoPE	−0.120	0.098	−0.262	–	0.027
	16,17-diHDoPE	0.041	0.579	−0.108	–	0.187
	19,20-diHDoPE	0.072	0.322	−0.075	–	0.216

PUFA, polyunsaturated fatty acid; 95% CI, 95% confidence interval; LA, linoleic acid; EpOME, epoxyoctadecamonoenoic acid; diHOME, dihydroxyoctadecenoic acid; AA, arachidonic acid; EET, epoxyeicosatrienoic acid; diHETrE, dihydroxyeicosaterolaenoic acid; EPA, eicosapentaenoic acid; EpETE, epoxyeicosatetraenoic acid; diHETE dihydroxyeicosatrienoic acid; DHA, docosahexaenoic acid; EpDPA, epoxydocosapentaenoic acid; diHDoPE, dihydroxydocosapentaenoic acid; N/A, not applicable.

### Cord blood fatty acid metabolite in SGA and AGA neonates

**Figure 1:**
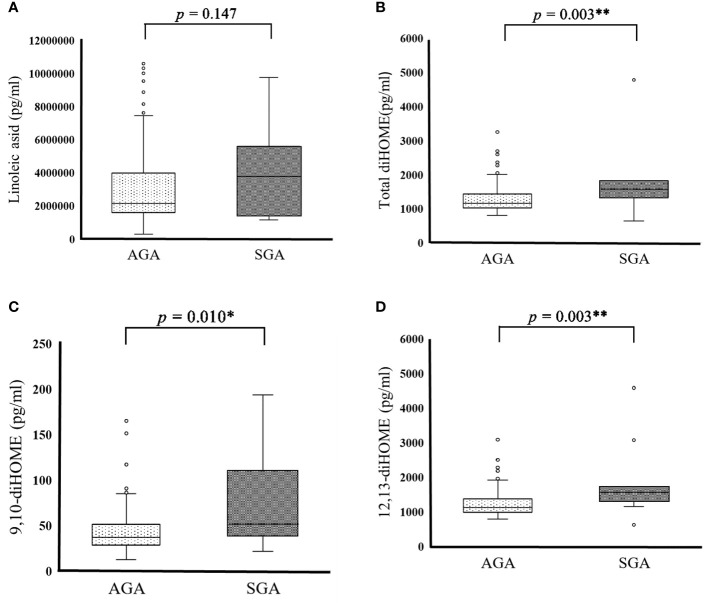
Cord blood PUFA metabolite in SGA and AGA infants. **(A)** Cord blood linoleic acid in AGA (2051991.877 ± 951304.536pg/mL) and SGA (2500019.845 ± 1318855.948 pg/mL) participants. **(B)** Cord blood total diHOME in AGA (1,272.320 ± 377.317 pg/mL) and SGA (1,915.757 ± 1,129.687 pg/mL) male subjects. **(C)** Cord blood 9, 10-diHOME in AGA (42.814 ± 22.070) and SGA (73.348± 49.116 pg/mL) participants. **(D)** Cord blood 12, 13-diHOME in AGA (1,229.506 ± 357.116 pg/mL) and SGA (1,842.409 ± 1,083.126 pg/mL) participants. Data are represented as the mean (± SD). Asterisks indicate **P < 0.01, *P < 0.05, Mann–Whitney *U* test, AGA (n = 179) and SGA (n = 11).

SGA and AGA are indicators that reflect placental and maternal problems in the second and third trimesters of pregnancy. We examined whether there are differences in PUFA and EpFA levels between SGA and AGA infants. PUFA and EpFA levels did not differ significantly between the SGA and AGA groups (**Table 6** and **Figure 1A**). Conversely, total diHOME (*p* = 0.003) (**Table 6** and **Figure 1B**), 9,10-diHOME (*p* = 0.010) (**Table 6** and **Figure 1C**), 12,13-diHOME (*p* = 0.003) (**Table 6** and **Figure 1D**), 5,6-diHETrE (*p* = 0.037) (**Table 6**), and 14,15-diHETE (*p* = 0.020) (**Table 6**) concentrations differed significantly between the SGA and AGA groups.

**Table 6:** Cord blood PUFA metabolite in SGA and AGA infants.

	SGA (n = 11)					AGA (n = 179)						
	Mean	SD	Range			Mean	SD	Range			Mann-Whitney *U* test	*p* value
**LA**	2.500 × 10^6^	1.319 × 10^6^	1.142 × 10^6^	–	4.631 × 10^6^	2.052 × 10^6^	0.951 × 10^6^	0.297 × 10^6^	–	4.953 × 10^6^	728.000	0.147
EpOME	0.891 × 10^3^	0.414 × 10^3^	0.510 × 10^3^	–	1.577 × 10^3^	1.127 × 10^3^	0.832 × 10^3^	0.399 × 10^3^	–	6.633 × 10^3^	962.000	0.899
9,10-EpOME	0.348 × 10^3^	0.228 × 10^3^	0.111 × 10^3^	–	0.718 × 10^3^	0.502 × 10^3^	0.453 × 10^3^	0.086 × 10^3^	–	3.546 × 10^3^	955.000	0.868
12,13-EpOME	0.543 × 10^3^	0.196 × 10^3^	0.314 × 10^3^	–	0.859 × 10^3^	0.624 × 10^3^	0.391 × 10^3^	0.214 × 10^3^	–	3.086 × 10^3^	926.000	0.741
diHOME	1.916 × 10^3^	1.130 × 10^3^	0.641 × 10^3^	–	4.774 × 10^3^	1.272 × 10^3^	0.377 × 10^3^	0.809 × 10^3^	–	3.246 × 10^3^	465.000	0.003
9,10-diHOME	0.733 × 10^2^	0.491 × 10^2^	0.211 × 10^2^	–	1.938 × 10^2^	0.428 × 10^2^	0.221 × 10^2^	0.119 × 10^2^	–	1.651 × 10^2^	526.500	0.010
12,13-diHOME	1.842 × 10^3^	1.083 × 10^3^	0.620 × 10^3^	–	4.580 × 10^3^	1.230 × 10^3^	0.357 × 10^3^	0.793 × 10^3^	–	3.081 × 10^3^	458.000	0.003
**AA**	2.275 × 10^5^	0.703 × 10^5^	1.443 × 10^5^	–	3.345 × 10^5^	2.266 × 10^5^	0.749 × 10^5^	0.868 × 10^5^	–	4.339 × 10^5^	950.000	0.845
EET	0.211 × 10^3^	0.278 × 10^3^	0.000	–	0.643 × 10^3^	0.462 × 10^3^	0.502 × 10^3^	0.000	–	3.792 × 10^3^	879.000	0.547
5,6-EET	0.523 × 10^2^	0.726 × 10^2^	0.000	–	1.640 × 10^2^	1.385 × 10^2^	1.832 × 10^2^	0.000	–	12.94 × 10^2^	882.000	0.555
8,9-EET	0.815 × 10^2^	1.046 × 10^2^	0.000	–	2.378 × 10^2^	1.745 × 10^2^	2.120 × 10^2^	0.000	–	10.64 × 10^2^	469.000	0.360
11,12-EET	0.358 × 10^2^	0.471 × 10^2^	0.000	–	1.022 × 10^2^	0.818 × 10^2^	0.881 × 10^2^	0.000	–	6.930 × 10^2^	673.000	0.501
14,15-EET	0.413 × 10^2^	0.837 × 10^2^	0.000	–	2.226 × 10^2^	0.668 × 10^2^	1.024 × 10^2^	0.000	–	7.405 × 10^2^	601.000	0.433
diHETrE	3.681 × 10^3^	0.947 × 10^3^	1.466 × 10^3^	–	4.926 × 10^3^	3.457 × 10^3^	0.950 × 10^3^	1.518 × 10^3^	–	6.697 × 10^3^	750.500	0.186
5,6-diHETrE	1.366 × 10^2^	0.259 × 10^2^	1.075 × 10^2^	–	1.567 × 10^2^	2.287 × 10^2^	1.251 × 10^2^	0.962 × 10^2^	–	8.975 × 10^2^	49.000	0.037
8,9-diHETrE	1.196 × 10^3^	0.533 × 10^3^	0.319 × 10^3^	–	2.081 × 10^3^	1.108 × 10^3^	0.406 × 10^3^	0.510 × 10^3^	–	2.369 × 10^3^	867.000	0.507
11,12-diHETrE	0.912 × 10^3^	0.300 × 10^3^	0.360 × 10^3^	–	1.506 × 10^3^	0.839 × 10^3^	0.292 × 10^3^	0.312 × 10^3^	–	1.841 × 10^3^	816.000	0.341
14,15-diHETrE	1.537 × 10^3^	0.401 × 10^3^	0.787 × 10^3^	–	2.054 × 10^3^	1.367 × 10^3^	0.396 × 10^3^	0.295 × 10^3^	–	2.574 × 10^3^	711.500	0.123
**EPA**	1.997 × 10^4^	0.787 × 10^4^	0.695 × 10^4^	–	2.985 × 10^4^	1.937 × 10^4^	0.751 × 10^4^	0.711 × 10^4^	–	5.297 × 10^4^	930.000	0.758
EpETE	0.000	0.000	0.000	–	0.000	0.052 × 10^2^	0.348 × 10^2^	0.000	–	2.602 × 10^2^	968.000	0.666
5,6-EpETE	0.000	0.000	0.000	–	0.000	0.000	0.000	0.000	–	0.000	476.000	1.000
8,9-EpETE	0.000	0.000	0.000	–	0.000	0.033 × 10^2^	0.268 × 10^2^	0.000	–	2.351 × 10^2^	469.000	0.747
11,12-EpETE	0.000	0.000	0.000	–	0.000	0.000	0.000	0.000	–	0.000	476.000	1.000
14,15-EpETE	0.000	0.000	0.000	–	0.000	0.000	0.000	0.000	–	0.000	476.000	1.000
17,18-EpETE	0.000	0.000	0.000	–	0.000	0.019 × 10^2^	0.224 × 10^2^	0.000	–	2.602 × 10^2^	472.500	0.821
diHETE	0.744 × 10^4^	0.348 × 10^4^	0.209 × 10^4^	–	1.391 × 10^4^	0.621 × 10^4^	0.310 × 10^4^	0.070 × 10^4^	–	2.990 × 10^4^	741.000	0.169
5,6-diHETE	0.000	0.000	0.000	–	0.000	0.000	0.000	0.000	–	0.000	N/A	N/A
8,9-diHETE	2.282 × 10^2^	0.119 × 10^2^	2.198 × 10^2^	–	2.367 × 10^2^	3.500 × 10^2^	1.502 × 10^2^	1.742 × 10^2^	–	8.121 × 10^2^	13.000	0.317
11,12-diHETE	1.424 × 10^2^	0.696 × 10^2^	0.335 × 10^2^	–	2.366 × 10^2^	1.233 × 10^2^	0.490 × 10^2^	4.182 × 10^2^	–	4.155 × 10^2^	437.500	0.253
14,15-diHETE	1.060 × 10^3^	0.598 × 10^3^	0.160 × 10^3^	–	2.225 × 10^3^	0.676 × 10^3^	0.244 × 10^3^	0.278 × 10^3^	–	1.450 × 10^3^	295.000	0.020
17,18-diHETE	6.528 × 10^3^	2.947 × 10^3^	1.674 × 10^3^	–	11.68 × 10^3^	5.552 × 10^3^	2.837 × 10^3^	2.180 × 10^3^	–	28.21 × 10^3^	725.000	0.149
**DHA**	8.873 × 10^5^	5.997 × 10^5^	2.657 × 10^5^	–	18.92 × 10^5^	8.941 × 10^5^	4.834 × 10^5^	0.223 × 10^5^	–	27.19 × 10^5^	893.000	0.605
EpDPA	0.201 × 10^3^	0.323 × 10^3^	0.000	–	0.807 × 10^3^	0.343 × 10^3^	0.612 × 10^3^	0.000	–	5.027 × 10^3^	816.500	0.340
4,5-EpDPA	0.000	0.000	0.000	–	0.000	0.000	0.000	0.000	–	0.000	476.000	1.000
7,8-EpDPA	0.339 × 10^2^	0.898 × 10^2^	0.000	–	2.373 × 10^2^	0.275 × 10^2^	1.006 × 10^2^	0.000	–	5.575 × 10^2^	463.000	0.719
10,11-EpDPA	0.503 × 10^2^	0.571	0.000	–	1.393 × 10^2^	1.207 × 10^2^	1.085 × 10^2^	0.000	–	6.363 × 10^2^	569.000	0.077
13,14-EpDPA	0.000	0.000	0.000	–	0.000	0.171 × 10^2^	0.876 × 10^2^	0.000	–	6.300 × 10^2^	498.000	0.282
16,17-EpDPA	0.277 × 10^2^	0.734 × 10^2^	0.000	–	1.941 × 10^2^	0.460 × 10^2^	1.300 × 10^2^	0.000	–	11.37 × 10^2^	444.000	0.624
19,20-EpDPA	0.894 × 10^2^	1.554 × 10^2^	0.000	–	3.633 × 10^2^	1.322 × 10^2^	3.625 × 10^2^	0.000	–	32.53 × 10^2^	492.500	0.867
diHDoPE	5.819 × 10^3^	1.512 × 10^3^	2.192 × 10^3^	–	7.691 × 10^3^	5.744 × 10^3^	1.941 × 10^2^	2.159 × 10^2^	–	15.46 × 10^2^	861.000	0.485
4,5-diHDoPE	N/A	N/A	N/A		N/A	N/A	N/A	N/A		N/A	N/A	N/A
7,8-diHDoPE	0.323 × 10^3^	0.112 × 10^3^	0.243 × 10^3^	–	0.402 × 10^3^	0.420 × 10^3^	0.274 × 10^3^	0.221 × 10^3^	–	2.074 × 10^3^	34.000	0.488
10,11-diHDoPE	2.202 × 10^2^	0.797 × 10^2^	1.058 × 10^2^	–	3.897 × 10^2^	2.587 × 10^2^	1.159 × 10^2^	0.581 × 10^2^	–	8.108 × 10^2^	780.000	0.280
13,14-diHDoPE	0.837 × 10^3^	0.232 × 10^3^	0.330 × 10^3^	–	1.159 × 10^3^	0.903 × 10^3^	0.320 × 10^3^	0.234 × 10^3^	–	1.830 × 10^3^	907.000	0.662
16,17-diHDoPE	0.631 × 10^3^	0.212 × 10^3^	0.262 × 10^3^	–	1.000 × 10^3^	0.649 × 10^3^	0.196 × 10^3^	0.222 × 10^3^	–	1.234 × 10^3^	852.000	0.843
19,20-diHDoPE	4.130 × 10^3^	1.160 × 10^3^	1.493 × 10^3^	–	5.417 × 10^3^	3.832 × 10^3^	1.341 × 10^3^	1.145 × 10^3^	–	11.28 × 10^3^	774.000	0.234

PUFA, polyunsaturated fatty acid; SGA, small for gestational age; AGA, appropriate for gestational age; SD, standard deviation; LA, linoleic acid; EpOME, epoxyoctadecamonoenoic acid; diHOME, dihydroxyoctadecenoic acid; AA, arachidonic acid; EET, epoxyeicosatrienoic acid; diHETrE, dihydroxyeicosaterolaenoic acid; EPA, eicosapentaenoic acid; EpETE, epoxyeicosatetraenoic acid; diHETE dihydroxyeicosatrienoic acid; DHA, docosahexaenoic acid; EpDPA, epoxydocosapentaenoic acid; diHDoPE, dihydroxydocosapentaenoic acid; N/A, not applicable.

### Correlation between birth weight and PUFA metabolite in cord blood after adjustment for potential confounding factors

After adjusting for obstetric factors, including gestational age at birth, infant’s sex, parity, delivery method (cesarean section), and maternal height, we found that birth weight was significantly correlated with LA concentration (*r =* −0.101, *p =* 0.016) (**Table 7**). Moreover, significant correlations were found between diHOME (*r =* −0.126, *p =* 0.007), 9,10-diHOME (*r =* −0.115, *p =* 0.014), and 12,13-diHOME (*r =* −0.126, *p =* 0.007) concentrations and birth weight (**Table 7**). No variables showed inflations in the variance inflation factor, indicating no multicollinearity with covariates.

**Table 7:** Correlation between birth weight and PUFA metabolite in cord blood after adjustment for potential confounding factors.

	*β*	non-standardized *β*	*r*	*p* value	95% CI			*R^2^*
**LA**	−0.149	−0.000022	−0.101	0.016	0.000046	–	0.00005	0.360
EpOME	0.035	0.018	0.070	0.564	−0.043	–	0.078	0.341
9,10 - EpOME	0.063	0.058	0.082	0.305	−0.053	–	0.169	0.343
12,13 - EpOME	0.003	0.003	0.053	0.967	−0.125	–	0.131	0.340
diHOME	−0.164	−0.129	−0.126	0.007	−0.221	–	−0.036	0.366
9,10 - diHOME	−0.149	−2.191	−0.115	0.014	−3.926	–	−0.456	0.361
12,13 - diHOME	−0.164	−0.135	−0.126	0.007	−0.232	–	−0.038	0.366
**AA**	0.044	0.000	0.031	0.478	0.000	–	0.001	0.341
EET	0.070	0.056	0.088	0.254	−0.400	–	0.152	0.344
5,6 − EET	0.109	0.244	0.127	0.074	−0.024	–	0.511	0.352
8,9 − EET	0.021	0.035	0.046	0.759	−0.189	–	0.258	0.346
11,12 − EET	0.055	0.239	0.046	0.396	−0.316	–	0.794	0.377
14,15 − EET	−0.015	−0.052	0.014	0.830	−0.529	–	0.425	0.355
diHETrE	−0.028	−0.011	−0.003	0.647	−0.058	–	0.036	0.342
5,6 - diHETrE	−0.035	−0.098	−0.051	0.651	−0.529	–	0.332	0.383
8,9 - diHETrE	−0.068	−0.061	−0.077	0.262	−0.168	–	0.046	0.344
11,12 - diHETrE	−0.011	−0.014	0.049	0.856	−0.167	–	0.139	0.340
14,15 - diHETrE	0.010	0.009	0.040	0.873	−0.103	–	0.121	0.340
**EPA**	−0.031	−0.002	0.018	0.609	−0.007	–	0.004	0.341
EpETE	0.049	0.622	0.127	0.422	−0.902	–	2.146	0.034
5,6 - EpETE	N/A	N/A	N/A	N/A	N/A		N/A	N/A
8,9 - EpETE	0.017	0.239	0.086	0.822	−1.731	–	2.210	0.368
11,12 - EpETE	N/A	N/A	N/A	N/A	N/A		N/A	N/A
14,15 - EpETE	N/A	N/A	N/A	N/A	N/A		N/A	N/A
17,18 - EpETE	0.054	0.915	0.112	0.443	−1.437	–	3.267	0.370
diHETE	−0.073	−0.009	−0.057	0.248	−0.023	–	0.006	0.344
5,6 - diHETE								
8,9 - diHETE	0.031	0.079	−0.016	0.886	−1.061	–	1.218	0.213
11,12 - diHETE	−0.103	−0.751	−0.051	0.131	−1.727	–	0.226	0.404
14,15 - diHETE	−0.124	−0.167	−0.091	0.055	−0.338	–	0.003	0.426
17,18 - diHETE	−0.068	−0.009	−0.053	0.280	−0.025	–	0.007	0.344
**DHA**	−0.071	−5.561	−0.086	0.243	0.000	–	0.000	0.345
EpDPA	−0.011	−0.007	−0.027	0.857	−0.079	–	0.066	0.340
4,5 - EpDPA	N/A	N/A	N/A	N/A	N/A		N/A	N/A
7,8 - EpDPA	−0.048	−0.172	−0.014	0.490	−0.663	–	0.319	0.367
10,11 - EpDPA	0.238	0.175	−0.033	0.462	−0.294	–	0.645	0.382
13,14 - EpDPA	−0.112	−0.432	−0.041	0.108	−0.959	–	0.096	0.037
16,17 - EpDPA	−0.006	−0.016	−0.004	0.934	−0.409	–	0.376	0.365
19,20 - EpDPA	−0.024	−0.024	−0.047	0.639	−0.123	–	0.075	0.365
diHDoPE	−0.030	−0.006	−0.028	0.636	−0.030	–	0.018	0.340
4,5 - diHDoPE	N/A	N/A	N/A	N/A	N/A		N/A	N/A
7,8 - diHDoPE	0.109	0.148	0.038	0.366	−0.179	–	0.475	0.465
10,11 - diHDoPE	−0.023	−0.075	−0.027	0.715	−0.482	–	0.331	0.339
13,14 - diHDoPE	−0.024	−0.029	−0.006	0.699	−0.174	–	0.117	0.340
16,17 - diHDoPE	−0.033	−0.062	−0.018	0.589	−0.290	–	0.165	0.341
19,20 - diHDoPE	−0.037	−0.010	−0.029	0.559	−0.044	–	0.024	0.341

PUFA, polyunsaturated fatty acid; 95% CI, 95% confidence interval; LA, linoleic acid; EpOME, epoxyoctadecamonoenoic acid; diHOME, dihydroxyoctadecenoic acid; AA, arachidonic acid; EET, epoxyeicosatrienoic acid; diHETrE, dihydroxyeicosaterolaenoic acid; EPA, eicosapentaenoic acid; EpETE, epoxyeicosatetraenoic acid; diHETE dihydroxyeicosatrienoic acid; DHA, docosahexaenoic acid; EpDPA, epoxydocosapentaenoic acid; diHDoPE, dihydroxydocosapentaenoic acid; N/A, not applicable.

## Discussion

Using the multiple regression analysis data from the HBC Study in Japan, we identified the significant association of birth weight with gestational age at birth, maternal height, infant’s sex, parity, delivery method (cesarean section), and umbilical cord serum LA concentration. After adjusting for obstetric confounding factors, birth weight showed a significant negative correlation with LA and diHOME concentrations in cord blood. To the best of our knowledge, the current study is the first to investigate the association of birth weight with EpFA and PUFA metabolism in cord blood.

Contrary to our hypothesis, birth weight showed no direct significant correlation with the concentration of any PUFA, EpFA, and diol (except for total EpETE) in cord blood before adjusting for obstetric confounding factors (**Table 3**). The result that EpETE is significantly correlated with birth weight could be false considering that there were multiple LLOD. After adjusting for obstetric confounding factors, such as gestational age at birth, infant’s sex, parity, delivery method (cesarean section), and maternal height, only LA and diHOME concentrations showed significant correlations with birth weight, indicating a specific relationship between birth weight and LA and its metabolism. In previous studies, ω3 and ω6 fatty acid concentrations in the maternal and cord blood have been found to be correlated (43), and a positive correlation was found between birth weight and erythrocyte fatty acid concentrations in the maternal and cord blood (44). In quantile regression analysis, birth weight was significantly associated with the maternal levels of LA, AA, and DHA and fetal levels of DHA in cord blood, but no significant correlation was noted for the fetal LA level (44). One possible explanation for this discrepancy is the difference in ethnic populations. Future studies are warranted to explain these inconsistent results.

The metabolism AA *via* the CYP pathway affects fetal development (18, 29, 33). However, we did not find any significant association between birth weight and AA or AA-derived EpFA concentrations in cord blood after adjusting for any obstetric confounding factor. We also investigated the effect of LA-derived EpOME and diHOME in fetal cord blood serum on the CYP metabolic pathway. LA is converted into LA epoxides, such as 9,10-epoxyoctadecenoic acid (9,10-EpOME) and 12,13-epoxyoctadecenoic acid (12,13-EpOME), by the CYP pathway (45) and is further metabolized into 9,10-DiHOME and 12,13-DiHOME by sEH (46). Both CYPs and sEHs have high substrate specificity, and CYP2J2, CYP2C8, and CYP2C9 are involved in LA metabolism (47). Interestingly, these CYP proteins are distributed in the placenta. IL-1β shows a significant positive correlation with CYP2J2 and CYP2C9 expressions in trophoblast cells, but it demonstrates a significant negative correlation with CYP2C8 expression in Hofbauer cells (20). Although we could not find a significant correlation between birth weight and any interleukin (including IL-1β) in cord blood in this study, the association between birth weight and LA or diHOME in the umbilical cord blood may be mediated by placental CYP2J2, CYP2C8, and CYP2C9.

EpOME influences the progression of acute and chronic inflammatory diseases (48, 49). sEH can bioactivate epoxides to diols, which are apparently cytotoxic. This toxicity is attributed to diHOME, and blocking the bioactivation reduces toxicity (50). EpOME and diHOME modulate vascular permeability and stimulate neutrophil chemotaxis (48). DiHOMEs are cardioprotective at low concentrations, but at higher levels, they have been implicated in vascular permeability and as cytotoxic agents and are associated with acute respiratory distress syndrome in patients with severe COVID-19 (51, 52). diHOME serum concentrations were significantly elevated in burn-injured mice; however, this elevation was reduced by the administration of 1-trifluoromethoxyphenyl-3-(1-propionylpiperidin-4-yl) urea, which is a sEH inhibitor (52). The inhibition of *in vivo* sEH can also block the toxicity of linoleate epoxides by stabilizing anti-inflammatory long-chain EpFAs and blocking the formation of the leukotoxin diols (53). Future research is required to examine the mechanism of action of LA and diHOME in cord blood on fetal development to clarify the promising therapeutic strategy of mitigating the deleterious outcomes of low-birth-weight.

Pregnancy has been associated with physiological inflammation, which involves the activation of the coagulation system, increased permeability of blood vessels, and production of inflammatory mediators, including AA and LA metabolites (34, 47). Miscarriage, preeclampsia, gestational diabetes, preterm delivery, and low-birth-weight can be caused by inflammation (32). The implantation of the fertilized egg results in the penetration of the blastocyst into the uterine mucosa, which “damages” the endometrium and replaces the uterine vasculature with a nutrient membrane; this replacement process requires an inflammatory environment for the uterus to induce structural and functional remodeling by mobilizing immune cells, such as macrophages, natural killer cells, and dendritic cells (35, 54). In the second trimester, during the anti-inflammatory phase, the fetus and mother have a symbiotic relationship, and both ω3 and ω6 fatty acids are actively transported to the fetus through the placenta and incorporated into the fetal tissues, red blood cells, and nervous system tissues (35). In the third trimester, an inflammatory state is again required, which is achieved with the infiltration of immune cells into the myometrium. The progressive detachment of the placenta from the uterine wall initiates the uterine contractions and delivery (35). In normal pregnancy, the previously mentioned AA and LA metabolites are usually involved in inflammatory responses and stress (18), but prostaglandin and thromboxane produced in the COX pathway are associated with fetal growth retardation (35). If the placenta is not functioning properly during this process, oxidative stress increases, and increased metabolism in the placenta and fetus also causes the production of reactive oxygen species. Normally, the balance between reactive oxygen species and intracellular antioxidants is maintained at appropriate levels, but once oxidative stress is prolonged, placental insufficiency, miscarriage, delayed fetal development, preeclampsia, and premature birth can occur (32). Given that the CYP pathway plays an important role in this process, the application of maternal anti-inflammatory interventional strategy during the third trimester may be necessary to avoid low-birth-weight.

### Limitations

This study has several limitations. First, the sample size was relatively small. In particular, the number of low-birth-weight and SGA infants was small; therefore, there may be a bias in the analysis of group differences. Second, we could not analyze the activities of CYP and sEH in cord blood because of limited research funding. Third, we have not collected maternal blood samples at childbirth. Therefore, we could not identify where the inflammation in cord blood came from in this study. Further studies on the association between LA and diHOME levels and birth weight in newborns *via* the bioactivation of placental CYP pathway needed to better understand the potential effects of LA and diHOME on fetal growth. Furthermore, epidemiological research is needed on possible mechanisms of action underlying the association between prenatal LA and diHOME exposure and fetal development. In addition, the potential effects of maternal LA and diHOME metabolism during pregnancy should be investigated.

## Conclusions

Birth weight was significantly correlated with LA and diHOME concentrations after adjusting for obstetric confounders, such as gestational age, maternal height, infant’s sex, childbirth history, and delivery method. However, this finding warrants further investigation on the placental CYPs. Furthermore, our study data showed that the LA and diHOME concentrations in cord blood potentially reflect intrauterine growth defects in infants. Hence, these findings may be useful in providing insights into the development of maternal intervention strategies to prevent low-birth-weight in infants.

## Data availability statement

The raw data supporting the conclusions of this article will be made available by the authors, without undue reservation.

## Ethics statement

The studies involving human participants were reviewed and approved by The Ethics Review Committee of Hamamatsu University School of Medicine and the Research Ethics Committee of University of Fukui. Written informed consent to participate in this study was provided by the participants’ legal guardian/next of kin.

## Author contributions

HM conceived and organized this study. NU analyzed the data and drafted the manuscript with the support of TH. TO-N performed the LC–MS/MS analysis. KT organized the HBC Study and supervised the present study. All the authors contributed to the discussion of the results and the creation of this manuscript.

## Funding

This work was supported, in part, by KAKENHI from the Ministry of Education, Culture, Sports, Science and Technology of Japan (19K21754 to HM, 22H00492 to KT). This work was also supported, in part, by Life Science Innovation Center, University of Fukui.

## Acknowledgments

We are grateful to the individuals who participated in the study. We would like to thank Ms. Fumiho Yamamoto and Ms. Natsuki Miyagoshi for technical assistance, Ms. Tomoko Taniguchi for clerical support, and Hiroki Nakanishi, PhD, from Lipidome Lab for providing technical advice for this study.

## Conflict of interest

Author TO-N was employed by company Lipidome Lab Co., Ltd.

The remaining authors declare that the research was conducted in the absence of any commercial or financial relationships that could be construed as a potential conflict of interest.

## Publisher’s note

All claims expressed in this article are solely those of the authors and do not necessarily represent those of their affiliated organizations, or those of the publisher, the editors and the reviewers. Any product that may be evaluated in this article, or claim that may be made by its manufacturer, is not guaranteed or endorsed by the publisher.

## References

[1] Wilcox AJ. On the importance–and the unimportance–of birthweight. Int J Epidemiol (2001) 30:1233–41. doi: 10.1093/ije/30.6.1233 11821313

[2] Khan A Deeba Nasrullah F Jaleel R. Frequency and risk factors of low birthweight in term pregnancy. Pak J Med Sci (2016) 32:138–42. doi: 10.12669/pjms.321.8120 PMC4795855 27022362

[3] Ganesh Kumar S Harsha Kumar HN Jayaram S Kotian MS. Determinants of low birth weight: A case control study in a district hospital in karnataka. Indian J Pediatr (2010) 77:87–9. doi: 10.1007/s12098-009-0269-9 19936646

[4] Ren X Vilhjálmsdóttir BL Rohde JF Walker KC Runstedt SE Lauritzen L. Systematic literature review and meta-analysis of the relationship between polyunsaturated and trans fatty acids during pregnancy and offspring weight development. Front Nutr (2021) 8:625596. doi: 10.3389/fnut.2021.625596 33842522 PMC8027310

[5] Kabaran S Besler HT. Do fatty acids affect fetal programming? J Health Popul Nutr (2015) 33:14. doi: 10.1186/s41043-015-0018-9 26825664 PMC5025983

[6] Delpisheh A Topping J Reyad M Tang A Brabin BJ. Prenatal alcohol exposure, CYP17 gene polymorphisms and fetal growth restriction. Eur J Obstet Gynecol Reprod Biol (2008) 138:49–53. doi: 10.1016/J.EJOGRB.2007.08.006 17875358

[7] Sasaki S Limpar M Sata F Kobayashi S Kishi R. Interaction between maternal caffeine intake during pregnancy and CYP1A2 C164A polymorphism affects infant birth size in the Hokkaido study. Pediatr Res (2017) 82:19–28. doi: 10.1038/pr.2017.70 28355205

[8] Vitti FP Grandi C Cavalli RC Simões VMF Batista RFL Cardoso VC. Association between caffeine consumption in pregnancy and low birth weight and preterm birth in the birth cohort of ribeirão preto. Rev Bras Ginecol Obstet (2018) 40:749–56. doi: 10.1055/s-0038-1675806 PMC10316898 30536269

[9] Soltani S Salari-Moghaddam A Saneei P Askari M Larijani B Azadbakht L. Maternal caffeine consumption during pregnancy and risk of low birth weight: a dose–response meta-analysis of cohort studies. Crit Rev Food Sci Nutr (2021) 1–10. doi: 10.1080/10408398.2021.1945532 34224282

[10] Hogervorst J Vesper HW Madhloum N Gyselaers W Nawrot T. Cord blood acrylamide levels and birth size, and interactions with genetic variants in acrylamide-metabolising genes. Environ Health (2021) 20:35. doi: 10.1186/s12940-021-00715-0 33794901 PMC8015021

[11] Windham GC Swan SH FL. Parental cigarette smoking and the risk of spontaneous abortion. Am J Epidemiol (1992) 135:1394–403. doi: 10.1093/oxfordjournals.aje.a116250 1510085

[12] Ward C Lewis S Coleman T. Prevalence of maternal smoking and environmental tobacco smoke exposure during pregnancy and impact on birth weight: Retrospective study using millennium cohort. BMC Public Health (2007) 7:81. doi: 10.1186/1471-2458-7-81 17506887 PMC1884144

[13] Günther V Alkatout I Vollmer C Maass N Strauss A Voigt M. Impact of nicotine and maternal BMI on fetal birth weight. BMC Preg Childbirth (2021) 21:127. doi: 10.1186/S12884-021-03593-Z PMC7881635 33579212

[14] Lindbohm ML Sallmén M Taskinen H. Effects of exposure to environmental tobacco smoke on reproductive health. Scand J Work Environ Health (2002) 28:84–96. 12058806

[15] Savitz DA Whelan EA Kleckner RC. Effect of parents’ occupational exposures on risk of stillbirth, preterm delivery, and small-for-gestational-age infants. Am J Epidemiol (1989) 129:1201–18. doi: 10.1093/oxfordjournals.aje.a115241 2729257

[16] Chand S Mustafa MD Banerjee BD Guleria K. CYP17A1 gene polymorphisms and environmental exposure to organochlorine pesticides contribute to the risk of small for gestational age. Eur J Obstet Gynecol Reprod Biol (2014) 180:100–5. doi: 10.1016/j.ejogrb.2014.06.016 25064838

[17] Cha J Sun X Dey SK. Mechanisms of implantation: Strategies for successful pregnancy. Nat Med (2012) 18:1754–67. doi: 10.1038/nm.3012 PMC6322836 23223073

[18] Kikut J Komorniak N Ziętek M Palma J Szczuko M. Inflammation with the participation of arachidonic (AA) and linoleic acid (LA) derivatives (HETEs and HODEs) is necessary in the course of a normal reproductive cycle and pregnancy. J Reprod Immunol (2020) 141:103177. doi: 10.1016/j.jri.2020.103177 32659532

[19] Cizkova K Tauber Z. Time-dependent expression pattern of cytochrome P450 epoxygenases and soluble epoxide hydrolase in normal human placenta. Acta Histochem (2018) 120:513–9. doi: 10.1016/J.ACTHIS.2018.06.002 29908721

[20] Tauber Z Chroma K Baranova R Cizkova K. The expression patterns of IL-1β and IL-10 and their relation to CYP epoxygenases in normal human placenta. Ann Anat- Anat Anz (2021) 236:151671. doi: 10.1016/J.AANAT.2020.151671 33440233

[21] Wu T Hu Y Chen C Yang F Li Z Fang Z. Passive smoking, metabolic gene polymorphisms, and infant birth weight in a prospective cohort study of Chinese women. Am J Epidemiol (2007) 166:313–22. doi: 10.1093/aje/kwm090 17526865

[22] Jump DB Depner CM Tripathy S. Omega-3 fatty acid supplementation and cardiovascular disease. J Lipid Res (2012) 53:2525–45. doi: 10.1194/jlr.R027904 PMC3494243 22904344

[23] Bazinet RP Layé S. Polyunsaturated fatty acids and their metabolites in brain function and disease. Nat Rev Neurosci (2014) 15:771–85. doi: 10.1038/nrn3820 25387473

[24] Layé S Nadjar A Joffre C Bazinet RP. Anti-inflammatory effects of omega-3 fatty acids in the brain: physiological mechanisms and relevance to pharmacology. Pharmacol Rev (2018) 70:12–38. doi: 10.1124/pr.117.014092 29217656

[25] Pu Y Yang J Chang L Qu Y Wang S Zhang K. Maternal glyphosate exposure causes autism-like behaviors in offspring through increased expression of soluble epoxide hydrolase. PNAS (2020) 117:11753–9. doi: 10.1073/pnas.1922287117/-/DCSupplemental PMC7260984 32398374

[26] Node K Huo Y Ruan X Yang B Spiecker M Ley K. Anti-inflammatory properties of cytochrome P450 epoxygenase-derived eicosanoids. NIH Public Access (1999) 20:1276–9. doi: 10.1126/science.285.5431.1276 PMC2720027 10455056

[27] McReynolds C Morisseau C Wagner K Hammock B. Epoxy fatty acids are promising targets for treatment of pain, cardiovascular disease and other indications characterized by mitochondrial dysfunction, endoplasmic stress and inflammation. Adv Exp Med Biol (2020) 1274:71–99. doi: 10.1007/978-3-030-50621-6_5 32894508 PMC7737916

[28] dos Santos LRB Fleming I. Role of cytochrome P450-derived, polyunsaturated fatty acid mediators in diabetes and the metabolic syndrome. Prostaglandins Other Lipid Mediat (2020) 148:106407. doi: 10.1016/j.prostaglandins.2019.106407 31899373

[29] Chang WC. Identification of an endogenous inhibitor of arachidonate metabolism in human epidermoid carcinoma A431 cells. J BioMed Sci (2003) 10:599–606. doi: 10.1159/000073525 14576462

[30] Dennis EA Norris PC. Eicosanoid storm in infection and inflammation. Nat Rev Immunol (2015) 15:511–23. doi: 10.1038/nri3859 PMC4606863 26139350

[31] Maybin JA Critchley HOD Jabbour HN. Inflammatory pathways in endometrial disorders. Mol Cell Endocrinol (2011) 335:42–51. doi: 10.1016/j.mce.2010.08.006 20723578

[32] Sultana Z Maiti K Aitken J Morris J Dedman L Smith R. Oxidative stress, placental ageing-related pathologies and adverse pregnancy outcomes. Am J Reprod Immunol (2017) 77:e12653. doi: 10.1111/aji.12653 28240397

[33] Calder PC Kremmyda L-S Vlachava M Noakes PS Miles EA. 3rd international immunonutrition workshop session 5: Early programming of the immune system and the role of nutrition is there a role for fatty acids in early life programming of the immune system? Proc Nutr Soc (2010) 69:373–80. doi: 10.1017/S0029665110001552 20462467

[34] Innes JK Calder PC. Omega-6 fatty acids and inflammation. Prostaglandins Leuko Essent Fatty Acids (2018) 132:41–8. doi: 10.1016/J.PLEFA.2018.03.004 29610056

[35] Szczuko M Kikut J Komorniak N Bilicki J Celewicz Z Ziętek M. The role of arachidonic and linoleic acid derivatives in pathological pregnancies and the human reproduction process. Int J Mol Sci (2020) 21:9628. doi: 10.3390/ijms21249628 PMC7766587 33348841

[36] Tsuchiya KJ Matsumoto K Suda S Miyachi T Itoh H Kanayama N. Searching for very early precursors of autism spectrum disorders: The hamamatsu birth cohort for mothers and children (HBC). J Dev Orig Health Dis (2010) 1:158–73. doi: 10.1017/S2040174410000140 25141784

[37] Takagai S Tsuchiya KJ Itoh H Kanayama N Mori N Takei N. Cohort profile: Hamamatsu birth cohort for mothers and children (HBC study). Int J Epidemiol (2016) 45:333–42. doi: 10.1093/ije/dyv290 26519951

[38] Hijioka M Futokoro R Ohto-Nakanishi T Nakanishi H Katsuki H Kitamura Y. Microglia-released leukotriene B4 promotes neutrophil infiltration and microglial activation following intracerebral hemorrhage. Int Immunopharmacol (2020) 85:106678. doi: 10.1016/J.INTIMP.2020.106678 32544870

[39] Ogawa Y Iwamura T Kuritani N Nishida H Takeuchi H Takada M. Birth size standards by gestational age for Japanese neonates. Acta Neonatologica Japonica (1998) 34:624–32.

[40] Voigt M Rochow N Landau-Crangle E Meyer-Kahrweg LM Olbertz DM Kunze M. Individualized sex-specific birth weight percentiles for gestational age based on maternal height and weight. J Perinat Med (2020) 49:94–103. doi: 10.1515/jpm-2020-0119 32866126

[41] Rahman MS Takahashi N Iwabuchi T Nishimura T Harada T Okumura A. Elevated risk of attention deficit hyperactivity disorder (ADHD) in Japanese children with higher genetic susceptibility to ADHD with a birth weight under 2000 g. BMC Med (2021) 19(1):229. doi: 10.1186/S12916-021-02093-3 34556092 PMC8461893

[42] Iwabuchi T Takahashi N Nishimura T Rahman MS Harada T Okumura A. Associations among maternal metabolic conditions, cord serum leptin levels, and autistic symptoms in children. Front Psych (2022) 12:816196. doi: 10.3389/fpsyt.2021.816196 PMC8851349 35185642

[43] Meher A Randhir K Mehendale S Wagh G Joshi S. Maternal fatty acids and their association with birth outcome: a prospective study. PloS One (2016) 11:e0147359. doi: 10.1371/journal.pone.0147359 26815428 PMC4729437

[44] Cinelli G Fabrizi M Ravà L Signore F Vernocchi P Semeraro M. Association between maternal and foetal erythrocyte fatty acid profiles and birth weight. Nutrients (2018) 10:402. doi: 10.3390/nu10040402 PMC5946187 29570689

[45] Reyes-Hernández CG Ramiro-Cortijo D Rodríguez-Rodríguez P Giambelluca S Simonato M González MDC. Effects of arachidonic and docosohexahenoic acid supplementation during gestation in rats. Implication placental Oxid stress Int J Mol Sci (2018) 19:3863. doi: 10.3390/ijms19123863 PMC6321355 30518038

[46] Newman JW Morisseau C Hammock BD. Epoxide hydrolases: their roles and interactions with lipid metabolism. Prog Lipid Res (2005) 44:1–51. doi: 10.1016/J.PLIPRES.2004.10.001 15748653

[47] Askari A Thomson SJ Edin ML Zeldin DC Bishop-Bailey D. Roles of the epoxygenase CYP2J2 in the endothelium. Prostaglandins Other Lipid Mediat (2013) 107:56–63. doi: 10.1016/j.prostaglandins.2013.02.003 23474289 PMC3711961

[48] Hildreth K Kodani SD Hammock BD Zhao L. Cytochrome P450-derived linoleic acid metabolites EpOMEs and DiHOMEs: A review of recent studies. J Nutr Biochem (2020) 86:108484. doi: 10.1016/j.jnutbio.2020.108484 32827665 PMC7606796

[49] Zheng J Plopper CG Lakritz J Storms DH Hammock BD. Leukotoxin-diol a putative toxic mediator involved in acute respiratory distress syndrome. Am J Respir Cell Mol Biol (2001) 25:434–8. doi: 10.1165/ajrcmb.25.4.4104 11694448

[50] Moghaddam MF Grant DF Cheek JM Greene F Williamson KC Hammock BD. Bioactivation of leukotoxins to their toxic dials by epoxide hydrolase. Nat Med (1997) 3:562–6. doi: 10.1038/nm0597-562 PMC7095900 9142128

[51] McReynolds CB Cortes-Puch I Ravindran R Khan IH Hammock BG Shih PB. Plasma linoleate diols are potential biomarkers for severe COVID-19 infections. Czechia Front Physiol (2021) 12:663869. doi: 10.3389/fphys.2021.663869 PMC8047414 33868029

[52] Bergmann CB Mcreynolds CB Wan D Singh N Goetzman H Caldwell CC. sEH-derived metabolites of linoleic acid drive pathologic inflammation while impairing key innate immune cell function in burn injury Proc Natl Acad Sci USA (2022) 119(13):e2120691119. doi: 10.1073/pnas.2120691119 35312372 PMC9060469

[53] Kodani SD Hammock BD. The 2014 Bernard b. brodie award lecture - epoxide hydrolases: Drug metabolism to therapeutics for chronic pain. Drug Metab Dispos (2015) 43:788–802. doi: 10.1124/dmd.115.063339 25762541 PMC4407705

[54] Mor G Cardenas I. The immune system in pregnancy: A unique complexity. Am J Reprod Immunol (2010) 63:425–33. doi: 10.1111/j.1600-0897.2010.00836.x PMC3025805 20367629

